# Influence of Tool-Axis Orientation on Dimensional Accuracy in Robot-Based Single Point Incremental Forming

**DOI:** 10.3390/ma19091761

**Published:** 2026-04-26

**Authors:** Alexandru Bârsan, Iosif-Adrian Maroșan, Sever-Gabriel Racz, Radu-Eugen Breaz, Mihai Crenganiș, Mihai-Octavian Popp, Gabriela-Petruța Popp, Diana-Maria Tatu

**Affiliations:** Department of Industrial Machines and Equipment, Engineering Faculty, Lucian Blaga University of Sibiu, Victoriei 10, 550024 Sibiu, Romania; alexandru.barsan@ulbsibiu.ro (A.B.); gabriel.racz@ulbsibiu.ro (S.-G.R.); radu.breaz@ulbsibiu.ro (R.-E.B.); mihai.crenganis@ulbsibiu.ro (M.C.); mihai.popp@ulbsibiu.ro (M.-O.P.); gabriela.popp@ulbsibiu.ro (G.-P.P.); diana.tatu@ulbsibiu.ro (D.-M.T.)

**Keywords:** single point incremental forming, robot-based incremental forming, tool-axis orientation, toolpath strategy, 3D scanning, dimensional accuracy

## Abstract

Single point incremental forming (SPIF) represents a flexible manufacturing process capable of producing complex sheet metal parts without the need for dedicated forming dies. However, achieving high dimensional accuracy remains a major challenge due to phenomena such as elastic springback and localized deformation. In this context, the present study investigates the influence of tool-axis orientation on the dimensional accuracy of parts manufactured through robot-based single point incremental sheet forming (RB-SPIF). The experimental analysis considered two toolpath strategies (contour and spiral), two vertical step sizes (0.5 mm and 1 mm), and two tool-axis configurations (fixed tool-axis and wall-normal tool-axis orientation), resulting in eight experimental cases. The dimensional accuracy of the manufactured parts was evaluated using optical 3D scanning and cross-sectional profile analysis. The results show that the vertical step size has a significant influence on the resulting geometry, with smaller step sizes generating profiles closer to the nominal geometry. The toolpath strategy also affects the geometry, with spiral trajectories generally producing slightly improved profiles compared to contour strategies; however, this effect was not found to be statistically significant under the investigated conditions. Furthermore, the use of a wall-normal tool-axis configuration improves the agreement between the measured and nominal profiles by enhancing the contact conditions between the tool and the metal sheet surface. These findings indicate that adaptive tool-axis orientation represents a promising strategy for improving the dimensional accuracy of parts produced by robot-based incremental sheet forming systems.

## 1. Introduction

Incremental Sheet Forming (ISF) has attracted increasing attention in recent decades due to its capability to manufacture complex sheet metal components without the need for dedicated forming dies. Compared with conventional forming processes such as deep drawing, stamping, or bending, ISF offers significant advantages including reduced tooling costs, shorter process preparation time, and enhanced geometric flexibility. These characteristics make the process particularly suitable for prototype manufacturing and small-batch production. As highlighted in recent review papers of the ISF process, the elimination of dedicated dies and the high adaptability of the process enable rapid manufacturing of customized components and complex geometries that would otherwise be difficult or uneconomical to produce using conventional forming methods [1,2,3,4].

**Figure 1:**
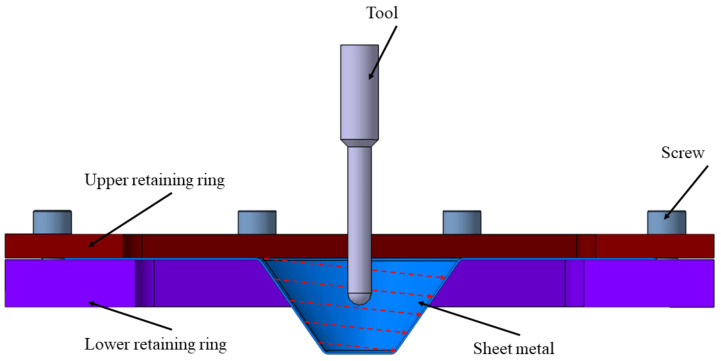
Single point incremental forming process [4].

Among the different variants of ISF, Single Point Incremental Forming (SPIF) is the most widely studied configuration [1,4]. In SPIF, the forming of the sheet metal blank is achieved through the localized and progressive action of a hemispherical or parabolic forming tool, known as punch, that follows a predefined toolpath over the workpiece surface, as shown in Figure 1. The sheet is typically clamped between a backing plate and a retaining frame while the punch gradually forms the material until the desired geometry is obtained. Owing to its relatively simple kinematic configuration and the absence of dedicated forming dies, SPIF has been extensively investigated in recent years as a flexible forming technology capable of producing complex geometries with reduced tooling requirements.

Despite these advantages, several limitations still limit the wider industrial implementation of SPIF. One of the most frequently reported drawbacks is the relatively low dimensional accuracy of the formed parts when compared with conventional forming processes. Previous studies have shown that geometric deviations may arise due to elastic springback, the localized nature of the deformation mechanism, and the accumulation of positioning errors along the toolpath [5,6,7,8,9,10,11]. In addition, the achievable wall angle is limited, and forming near-vertical walls remains particularly challenging. Consequently, improving the dimensional accuracy of SPIF parts has become one of the most important challenges in this field.

From a technological point of view, the SPIF process can be implemented on several types of equipment, including dedicated forming machines, CNC milling machines, and industrial robots [12,13]. Generally, SPIF is performed on CNC machine tools because of their high structural rigidity and good positioning accuracy. However, CNC-based systems also present certain limitations, particularly regarding workspace size and kinematic flexibility. In conventional three-axis CNC configurations, the orientation of the forming tool is typically kept constant throughout the process, which restricts the possibility of implementing more advanced forming strategies involving adaptive tool orientations.

In recent years, industrial robots have emerged as an alternative platform for incremental forming applications. Robots provide a large working envelope and high kinematic flexibility due to their six degrees of freedom, enabling both tool positioning and continuous adjustment of its orientation relative to the workpiece surface. Several studies have shown that robot-based incremental sheet forming (RB-SPIF) systems can be successfully used for manufacturing complex components while offering advantages in terms of flexibility and reduced investment costs compared with multi-axis CNC machines [13,14,15].

Nevertheless, robotic systems also introduce specific challenges, mainly related to their lower structural stiffness compared to CNC machines. Due to their serial kinematic structure, industrial robots are more prone to positioning errors caused by elastic deformations under forming forces. In addition, the loading conditions in SPIF differ from those encountered in conventional machining operations, which may further amplify these deviations. As a result, the dimensional accuracy obtained in RB-SPIF may be lower than in CNC-based forming. Therefore, the influence of robot stiffness and process loading conditions remains an important aspect in the analysis of RB-SPIF processes, while compensation strategies are still under investigation to improve accuracy [6,16].

Several recent studies have quantified the limitations and potential improvements of RB-SPIF systems. Palwai et al. [17] demonstrated that, for a hemispherical component with a wall angle of 90°, the maximum achievable forming depth increased from 11.75 mm in single-stage forming to 30 mm when using a preform-based multi-stage strategy, corresponding to an improvement of approximately 155%. Furthermore, the use of intermediate preforms resulted in a more uniform thickness distribution and delayed the onset of fracture. These results are particularly relevant in the context of robot-based incremental sheet forming (roboforming), where the inherent flexibility of industrial robots enables the implementation of complex multi-stage strategies and adaptive toolpaths that are difficult to achieve using conventional CNC systems. Experimental studies conducted by Singh et al. [18] on robot-assisted incremental sheet forming under hydrostatic support conditions have shown that process modifications can significantly improve thickness distribution and reduce springback. For instance, using a six-axis industrial robot with a spiral toolpath and a step size of 0.6 mm, parts with wall angles up to 40–43° were successfully formed at tool speeds of 200 mm/s. The application of back hydrostatic pressure reduced springback from 11% to 4% in conical geometries and from 5.3% to 2.22% in pyramidal shapes, while also increasing the region of uniform thickness along the formed wall. Breaz et al. [13] highlight that industrial robots used in incremental forming processes exhibit lower positional accuracy compared to CNC machine tools due to their serial kinematic structure. The cumulative errors from each joint, combined with reduced structural stiffness, lead to deviations of the tool trajectory under forming forces. However, industrial robots remain a viable solution for SPIF applications due to their high flexibility, large workspace, and ability to generate complex toolpaths. The authors emphasize that the performance of robot-based forming systems strongly depends on process parameters, machine configuration, and control strategies. Scholz et al. [15] propose a hybrid approach combining real-time optical sensing with ultrasonic vibration assistance to enhance both dimensional accuracy and process efficiency. Their study demonstrates that a shadow imaging sensor network can achieve tool position tracking with uncertainties below 50 µm, enabling effective compensation of robot trajectory deviations during forming. Additionally, the integration of ultrasonic vibrations significantly reduces forming forces—by up to 70%—while also improving friction conditions and minimizing springback effects. These findings highlight the potential of combining advanced sensing technologies with vibration-assisted forming to improve the reliability and industrial applicability of robot-based ISF processes.

The performance of the SPIF process is strongly influenced by several process parameters. Among the most widely investigated parameters are the tool diameter, feed rate, lubrication conditions, vertical step size, and toolpath strategy [3,4,19]. In particular, the type of toolpath plays a significant role in determining the distribution of deformation and the final geometry of the formed component. Two commonly used strategies are contour toolpaths, where the tool follows successive circular trajectories at different depth levels, and spiral toolpaths, which provide a continuous tool movement along the forming surface. Vanhulst et al. [9] investigated the influence of toolpath strategies on the geometric accuracy of SPIF parts and reported that continuous spiral trajectories lead to smoother thickness transitions and lower profile deviations compared with discrete contour strategies, particularly in multi-stage and complex geometries. Similarly, Jung et al. [10] demonstrated that optimized toolpath design can significantly decrease shape error by reducing local stress accumulation at transition zones. These findings support the idea that the continuity of the spiral trajectory reduces abrupt changes in loading direction and minimizes local force peaks, which directly contributes to lower springback and improved dimensional fidelity. Recent ESAFORM benchmark studies further confirmed that trajectory continuity is one of the dominant factors controlling geometric deviations in SPIF, especially in complex shapes where local deformation history strongly affects the final geometry [20].

For complex geometries, however, conventional contour and spiral toolpaths are often not sufficient to ensure high dimensional accuracy. Recent studies have increasingly focused on improving dimensional accuracy in SPIF through advanced toolpath strategies and adaptive kinematics. Duflou et al. [1], in their state-of-the-art review, emphasized that dimensional accuracy remains one of the major unresolved limitations of SPIF and highlighted advanced trajectory planning and compensation as critical research directions for further development of the process. Lu et al. [6] further showed that model predictive control can significantly improve geometric accuracy by compensating for process-induced deviations during forming, while Behera et al. [8] proposed adaptive compensation strategies based on regression models to reduce shape errors. These approaches highlight the need for adaptive toolpath design when dealing with complex SPIF geometries.

In the context of robotic incremental forming, several authors highlighted that tool orientation plays a critical role in contact mechanics. Mohanty et al. [21] showed that aligning the tool axis with the local surface normal improves material flow and reduces localized thinning. Moreover, Vanhulst et al. [20] emphasized that combined optimization of toolpath and tool orientation is essential for achieving high geometric accuracy in SPIF components. Despite these advances, limited studies systematically investigate the combined influence of tool-axis orientation, toolpath strategy, and vertical step size in robot-based SPIF systems, which motivates the present work.

Another critical parameter is the vertical step size, which directly influences both dimensional accuracy and processing time, as it controls the magnitude of deformation applied at each forming increment. Previous studies [3,4,20] have shown that smaller step sizes lead to improved dimensional accuracy and smoother surface profiles, while larger step sizes tend to increase geometric deviations due to higher deformation increments. For example, in the ESAFORM benchmark study on complex SPIF geometries, it was reported that reducing the step size leads to lower geometric deviations in the wall and transition regions because the strain is distributed more progressively over successive tool passes [20]. These results highlighted that larger step sizes amplify the deformation history between consecutive tool positions, which increases elastic recovery after unloading and consequently worsens dimensional accuracy. Similarly, Mohanty et al. [21] investigated robot-assisted incremental sheet forming under different forming conditions and observed that smaller vertical increments improve wall quality and reduce springback-related profile errors, particularly in conical benchmark parts. The authors attributed this behavior to the reduction in local bending severity and more stable material flow during forming. A comparable trend was also reported by Tera et al. [22], who showed from a CAM-oriented perspective that step size selection directly affects the discretization quality of the generated toolpath and the resulting geometric fidelity of SPIF parts. Their study emphasized that smaller steps improve approximation of the nominal CAD surface, leading to smoother trajectories and better adherence to the target geometry. In addition, larger step sizes have been associated with increased residual stresses, springback, and the pillow effect, all contributing to reduced geometric accuracy. Smaller step sizes generally enhance surface finish and reduce springback, although they also increase the total forming time. Instead, larger step sizes may enhance productivity but often result in increased geometric deviations and reduced part accuracy. Typical step sizes reported in the literature for high-accuracy SPIF range between 0.2 mm and 0.6 mm [20,21,22,23,24,25,26].

The issue of dimensional accuracy has been extensively investigated in recent years through both experimental and numerical studies. A recent benchmark study conducted within the ESAFORM community provided a comprehensive evaluation of geometric deviations in SPIF processes and identified dimensional accuracy as one of the main limitations of incremental forming technologies [20]. The authors reported that deviations frequently occur in regions such as inclined walls, bottom areas of the formed part, and transition zones between surfaces. Furthermore, the study emphasized that process strategies—including toolpath generation and process parameter selection—can significantly influence the resulting geometric deviations. Based on these observations, several research directions have been proposed for improving SPIF accuracy, including the optimization of toolpath strategies, the use of numerical simulations for deviation prediction, and the development of adaptive forming strategies.

Building on these findings, recent research has primarily focused on optimizing toolpath generation methods and process parameters to improve forming accuracy. Mohanty et al. [23] investigated the influence of part inclination and rotational motion in robot-assisted incremental sheet forming (RAISF) in order to improve forming quality and extend the achievable wall angles. In their approach, a robotic manipulator was used to orient the sheet metal part relative to the forming tool, enabling controlled tilting of the workpiece during the forming process. The results showed that the inclination of the part significantly affects the contact conditions between the tool and the sheet, leading to improvements in surface quality and allowing the formation of steeper wall angles compared with conventional SPIF. The study demonstrated that the additional degrees of freedom provided by robotic systems enable alternative forming strategies, such as part tilting, which can enhance process flexibility and improve forming performance. These findings highlight the potential of controlled inclination in robotic incremental forming as a promising approach for improving both dimensional accuracy and surface integrity of formed parts. However, in most of the existing studies the orientation of the forming tool axis is maintained as constant during the forming process. When industrial robots are used as forming platforms, the additional kinematic degrees of freedom enable the tool orientation to be adjusted relative to the local geometry of the part, for example by aligning the tool axis with the local surface normal.

In this context, the present study investigates the influence of tool-axis orientation on the dimensional accuracy of parts produced through RB-SPIF. Two commonly used toolpath strategies—circular (contour) and spiral trajectories—are analyzed in combination with two vertical step sizes. For each configuration, the forming process was performed both with a fixed tool-axis orientation and with the tool axis oriented perpendicular to the local wall of the formed part. The experimental results allow a comparative evaluation of the influence of these parameters on the geometric deviations of the formed components.

## 2. Materials and Methods

This section presents the materials, equipment, and experimental procedures used to investigate the influence of tool-axis orientation on the dimensional accuracy of parts manufactured through RB-SPIF. The experimental methodology includes the description of the robotic setup, the characteristics of the sheet blank and workpiece geometry, the strategy used for toolpath generation, and the experimental design adopted for the study.

The forming trajectories were generated offline using a dedicated CAM environment and subsequently implemented on an industrial robotic system equipped with a custom tool-holder unit and a hemispherical punch. The experimental strategy consisted of multiple forming experiments performed under different combinations of toolpath strategies, vertical step sizes, and tool-axis orientations.

After the forming process, the dimensional accuracy of the produced parts was evaluated using optical 3D scanning. The digitized geometries of the formed parts were aligned with the nominal CAD model of the component, allowing the generation of deviation maps and quantitative assessment of dimensional deviations.

This experimental approach enables a comparative investigation of the influence of toolpath strategy, vertical step size, and tool-axis orientation on the dimensional accuracy of parts produced by RB-SPIF.

### 2.1. Experimental Setup of the RB-SPIF

**Figure 2:**
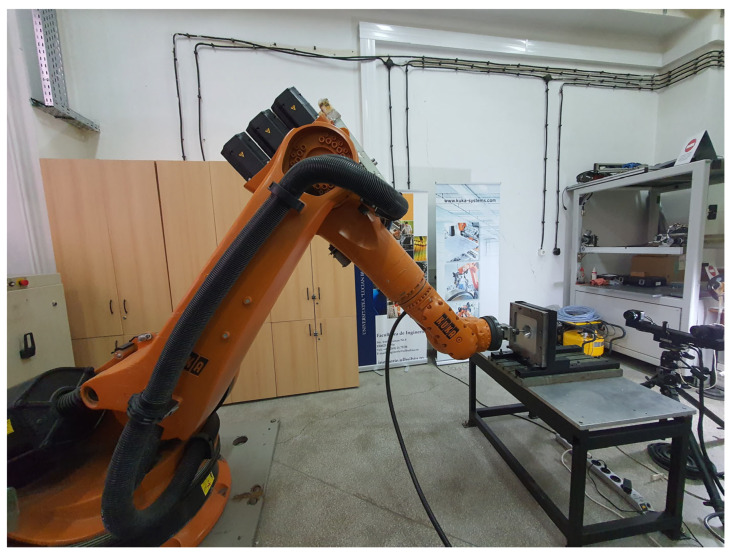
Experimental setup of the robot-based incremental sheet forming system.

The experiments were performed using a robotic cell designed for robot-based incremental sheet forming, as shown in Figure 2. The experimental setup consists of an industrial robot with six degrees of freedom, a dedicated tool holder mounted on the robot flange, a hemispherical punch, and a clamping system used to fix the sheet blank during the forming process.

The forming operations were performed using an industrial serial robot KUKA KR 210-2 (KUKA Deutschland Gmbh, Augsburg, Germany), which is widely used in industrial applications requiring large workspace and high load capacity. The robot provides six degrees of freedom, enabling both positional and orientational control of the punch during the forming process. This characteristic is particularly important for SPIF processes where the orientation of the forming tool relative to the sheet surface may significantly influence the forming mechanics and the final dimensional accuracy of the part. Industrial robots are known to exhibit lower structural stiffness compared to conventional CNC machines, which may lead to positioning errors due to elastic deformations under forming forces. However, because the purpose of the present work is a comparative evaluation of process parameter influence rather than absolute machine-tool benchmarking, all experiments were intentionally conducted under identical robot configuration, clamping conditions, tool geometry, and feed conditions. Under these controlled conditions, the compliance-related deviations of the serial robotic structure can be considered systematic and repeatable across all experimental cases, enabling a valid relative comparison between the investigated strategies. This assumption is consistent with previous RB-SPIF studies where comparative analyses were successfully conducted under constant robotic loading conditions.

The robot is controlled by the KUKA KR C2 controller(KUKA Deutschland GmbH, Augsburg, Germany). The trajectories followed by the punch are generated offline using a ENCY software (ENCY v.1, Limassol, Cyprus) and then imported into the robot control system. Offline programming allows accurate definition of the tool trajectory and simplifies the implementation of different forming strategies.

The sheet blank is fixed using a clamping device consisting of a rigid backing plate and a retaining frame. The clamping system ensures that the sheet remains rigidly fixed during the forming process and prevents unwanted displacements of the blank during process. Maintaining a stable clamping configuration is essential in incremental forming because even small displacements of the blank can influence the final geometry of the formed part.

The forming tool used in the experiments consists of a hemispherical punch made from hardened steel. The tool has a spherical tip with a diameter of 8 mm, which is a common configuration used in SPIF experiments [25,26]. The hemispherical geometry of the punch allows smooth contact with the sheet surface and reduces the risk of localized damage during the forming process.

**Figure 3:**
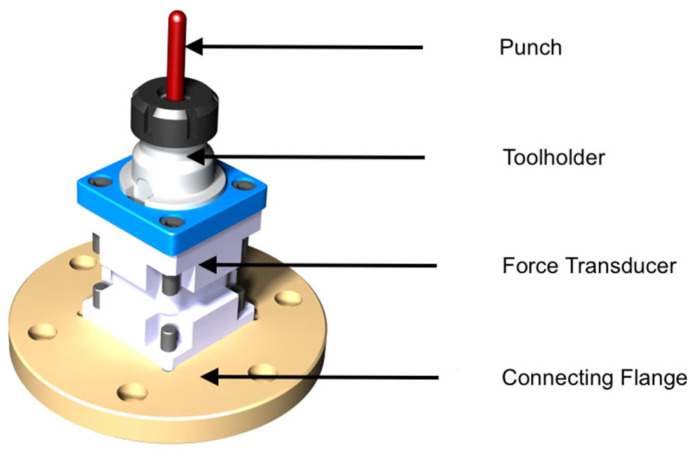
Tool holder unit used for the RB-SPIF experiments.

The punch mounted on a dedicated tool-holder unit specifically designed for robot-based incremental sheet forming applications, is presented in Figure 3. The tool holder is attached directly to the robot flange and ensures rigid positioning of the tool during forming. The tool-holder assembly allows the punch to transmit the forming forces generated during the process while maintaining precise orientation control [14,15].

The robotic configuration enables full control of both the position and orientation of the punch, which makes it possible to implement forming strategies involving variable tool orientation. This capability represents one of the main advantages of using robotic systems instead of conventional CNC machines for incremental forming processes.

During the forming process, the robot moves the punch along a predefined trajectory over the sheet surface. The progressive contact between the punch and the sheet blank generates localized plastic deformation, which gradually transforms the initially flat sheet into the desired three-dimensional shape.

### 2.2. Workpiece Material and Geometry

The experimental investigations were carried out using sheet blanks made from DC04 deep drawing steel, with a thickness of 0.67 mm, a material commonly used in sheet metal forming applications due to its good formability and ductility [22]. The selection of material and its mechanical properties have a direct influence on springback and geometric accuracy, which are key aspects analyzed in this study.

The mechanical properties of the material were determined experimentally through uniaxial tensile tests in accordance with ISO 6892-1:2020 [27]. The uniaxial tensile tests were performed using an Instron 5587 testing machine (Instron, Norwood, MA, USA). The testing procedure was controlled using Bluehill 2 software, which provides automatic calibration, system monitoring, and real-time acquisition of force and displacement data. Based on the recorded data, the key mechanical properties of the material were determined, including yield strength, ultimate tensile strength, and elongation at fracture. The average values obtained from the tests are: Young’s modulus of approximately 62,589 MPa, yield strength of 161.41 MPa, ultimate tensile strength of 289.98 MPa, and elongation at break of 27.66%. The mechanical proprieties of the DC04 steel are presented in Table 1.

The material exhibits typical behavior of low-carbon deep drawing steels, with good formability and moderate strain hardening (*n* ≈ 0.246), making it suitable for incremental forming processes. These properties play a key role in the springback behavior and dimensional accuracy of the formed parts.

The sheet blanks were cut into square specimens in order to ensure proper clamping in the forming fixture system. During the forming process, the sheet blank was rigidly clamped between a backing plate and a retaining frame to prevent undesired movements during forming process.

The geometry selected for the experimental investigations corresponds to a truncated cone-shaped part, which represents a commonly used benchmark geometry in incremental sheet forming research [20]. Such geometries are frequently adopted in SPIF studies because they allow the investigation of forming mechanisms and geometric deviations along inclined walls.

The main geometric parameters of the part are summarized in Table 2.

**Figure 4:**
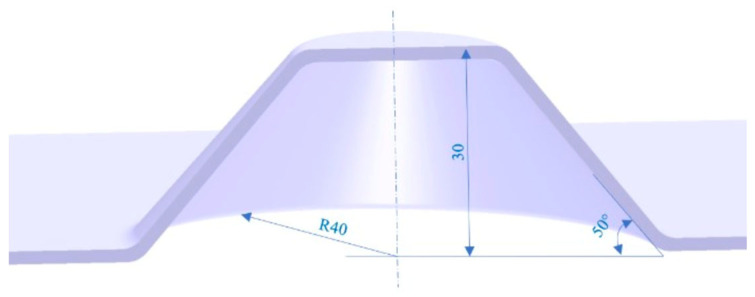
CAD model of the benchmark part used in the experiments.

The CAD model of the target geometry used for toolpath generation is illustrated in Figure 4.

### 2.3. Toolpath Generation and Tool-Axis Orientation Strategy

The toolpaths used for the incremental forming experiments were generated offline using a ENCY software. The toolpaths were generated based on the CAD model of the target part geometry. Two main toolpath strategies commonly used in SPIF processes were considered in the present study:

- Contour toolpath strategy;
- Spiral toolpath strategy.

In the contour toolpath strategy, the punch moves along a series of circular trajectories corresponding to different depth levels. Each contour represents a constant vertical level, and the tool moves gradually downward by a predefined vertical step between successive contours.

This strategy is widely used in incremental forming because it provides a clear discretization of the forming steps [8,9,10]. However, the discontinuous transition between successive contours may sometimes lead to small surface irregularities or deviations in the formed geometry.

In the spiral toolpath strategy, the punch follows a continuous spiral trajectory that gradually moves downward while progressing along the surface of the part. Unlike the contour strategy, the spiral trajectory does not involve discrete depth levels, which results in a smoother transition between successive forming stages. The use of spiral toolpaths is often associated with improved surface quality and smoother deformation transitions compared with contour strategies, leading to a more uniform strain distribution and reduced local stress concentrations [6,7,8,9,10,11]. In this study, simple toolpath strategies were preferred to isolate and clearly evaluate the influence of individual process parameters.

In addition to the toolpath strategy, the orientation of the forming tool axis was considered as an important parameter in the present study.

Two tool-axis orientation strategies were investigated:

- Fixed tool-axis orientation

In this configuration, the axis of the forming tool remains perpendicular to the initial plane of the sheet blank during the entire forming process. This approach is commonly used in conventional SPIF processes performed on CNC milling machines.

- Wall-normal tool-axis orientation

In this configuration, the tool axis is oriented perpendicular to the local surface of the part wall. This strategy requires continuous adjustment of the punch orientation along the toolpath, which can be implemented using the additional degrees of freedom available in robotic systems.

The wall-normal orientation strategy allows the punch to remain aligned with the local surface normal of the part geometry, which may improve the contact conditions between the tool and the sheet material and potentially lead to improved dimensional accuracy.

**Figure 5:**
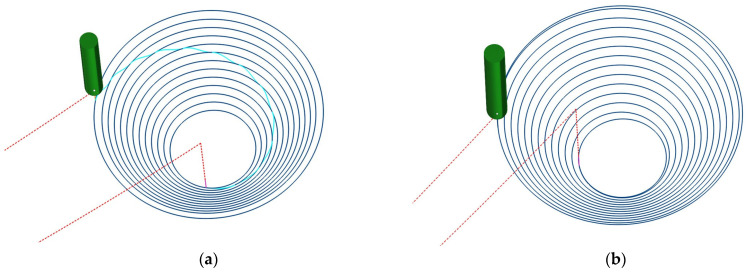

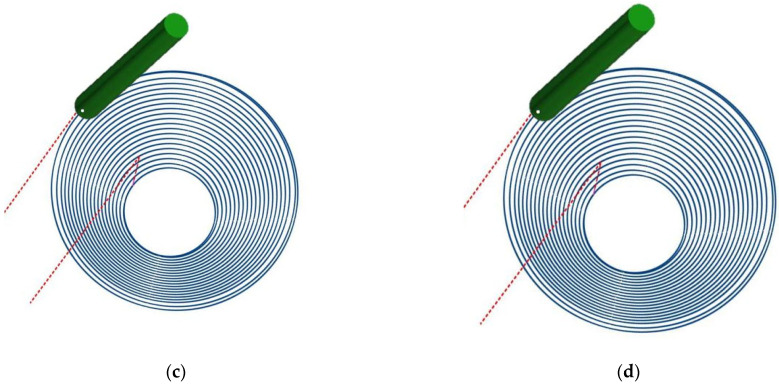
Tool-axis orientation strategies: (**a**) Contour toolpath strategy—fixed orientation; (**b**) Spiral toolpath strategies—fixed orientation; (**c**) Contour toolpath strategy—wall-normal orientation; (**d**) Spiral toolpath strategies—wall-normal orientation.

The tool-axis orientation strategies are presented in Figure 5.

In order to define the investigated tool-axis strategies in a rigorous and reproducible manner, the orientation of the punch was described with respect to the global Cartesian reference frame OXYZ, where the Z-axis is normal to the initial plane of the sheet blank. Two tool-axis configurations were considered in the present study, namely a fixed tool-axis orientation and a wall-normal tool-axis orientation associated with the wall angle of the nominal geometry.

In the fixed tool-axis configuration, the axis of the forming tool remains parallel to the global vertical direction during the entire forming process. The corresponding unit vector of the tool axis is therefore given by:

(1) $$a_{f}=\left[\begin{array}{c} 0\\ 0\\ 1 \end{array}\right],$$

In the wall-normal tool-axis configuration, the inclination of the tool was prescribed as a function of the wall angle of the nominal part geometry. Since the investigated part has a truncated-cone geometry, the wall angle remains constant and equal to α, whereas the angular position of the tool varies continuously along the toolpath. Under these conditions, the unit vector describing the wall-normal tool-axis orientation may be written as:

(2) $$a_{i}(\theta )=\left[\begin{array}{c} \sin\alpha \;\cos\;\theta \\ \sin\alpha \;\sin\;\theta \\ \cos\alpha \end{array}\right],$$

where α represents the nominal wall angle and θ represents the instantaneous angular position of the tool along the forming trajectory.

According to this interpretation, the magnitude of the tool-axis inclination is determined by the wall angle of the part, while the spatial direction of the axis is continuously updated as a function of the tool position along the trajectory. In this way, the fixed strategy corresponds to a constant tool-axis orientation, whereas the wall-normal strategy corresponds to a variable orientation adapted to the wall geometry of the part.

### 2.4. Experimental Planning and Dimensional Accuracy Evaluation

To evaluate the influence of the investigated parameters on the dimensional accuracy of the formed parts, a structured experimental plan was developed.

Two vertical step sizes were selected for the experimental study: 0.5 mm and 1 mm. The smaller value (0.5 mm) was selected as a reference high-accuracy forming condition, based on commonly reported values in the literature [4,9,19], while the larger value (1 mm) was included to evaluate the influence of step size and to assess the trade-off between dimensional accuracy and process efficiency.

By combining the two toolpath strategies, the two step size values, and the two tool-axis orientation strategies, a total of eight experimental cases were defined. The experimental plan is summarized in Table 3.

Each experimental configuration was performed using the robotic incremental forming system described previously. The forming experiments were carried out under identical processing conditions in order to minimize experimental variability and ensure the repeatability of the results.

**Figure 6:**
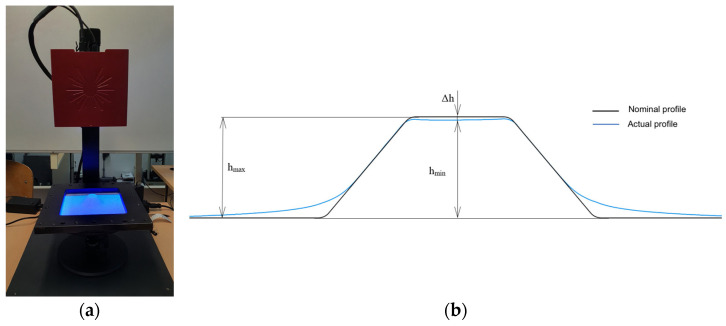
Dimensional accuracy evaluation of the formed parts: (**a**) ATOS Core 200 optical system; (**b**) Comparison between the nominal and measured profiles.

After the forming process, the dimensional accuracy of the produced parts was evaluated using optical 3D scanning [28]. The surface of each formed part was digitized using the ATOS Core 200 optical scanning system (Carl Zeiss GOM Metrology GmbH, Braunschweig, Germany), as presented in Figure 6. During the scanning process, calibrated reference markers with a diameter of 1.5 mm were applied on the surface of the part that was not in contact with the punch in order to improve the accuracy of the scanning procedure.

The scanning process generated dense point clouds representing the geometry of the formed parts. The acquired point clouds were processed and analyzed using the GOM Inspect metrology software (GOM Inspect v2020, Braunschweig, Germany). The scanned geometries were aligned with the nominal CAD model of the part, allowing the generation of deviation maps that highlight the geometric differences between the formed parts and the target geometry.

The analysis focused on evaluating the influence of the main process parameters on the dimensional accuracy of the formed parts, namely:

- Vertical step size;
- Toolpath strategy;
- Tool-axis orientation.

**Figure 7:**
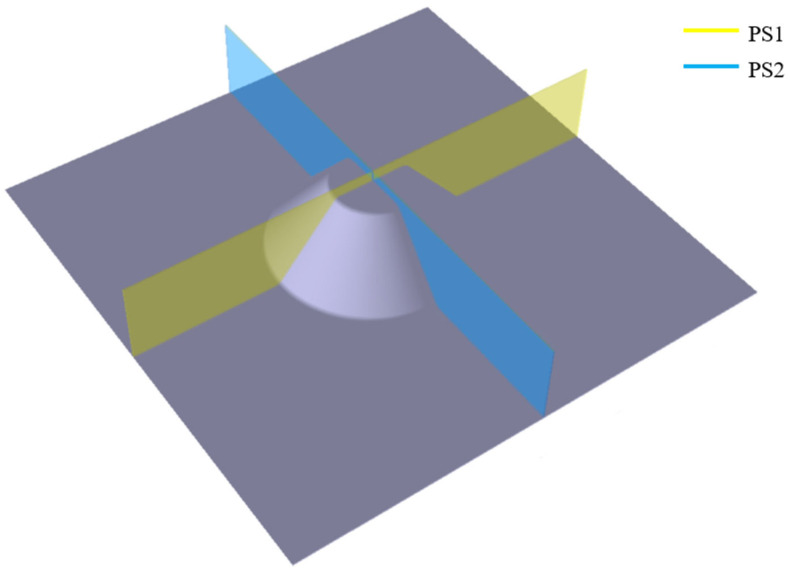
Cross-sectional planes used for dimensional accuracy evaluation: PS1 (0°), PS2 (90°).

In order to perform a detailed geometric analysis, two cross-sectional profiles were extracted for each formed part, as shown in Figure 7. These profiles were obtained by intersecting the scanned geometry with two orthogonal transverse planes, denoted PS1 and PS2.

The first plane, PS1, is oriented at 0° relative to the rolling direction of the sheet, while the second plane, PS2, is oriented along the transverse direction at 90° relative to the rolling direction. Both planes pass through the axis of the conical wall of the formed part, allowing the evaluation of geometric deviations along representative sections of the component.

The obtained contour curves were compared with the nominal reference circle obtained by the CAD model. This comparison enabled the evaluation of form deviations and the assessment of the circularity of the formed geometry at the selected height.

## 3. Results

The dimensional accuracy of the parts produced by RB-SPIF was evaluated by comparing the scanned geometry of the parts with the nominal CAD model. The analysis was performed using the cross-sectional profiles extracted along the planes PS1 and PS2, as shown in Figure 7. These sections allowed the evaluation of both dimensional deviations along the wall profile and form deviations of the part at a constant height.

The results obtained for the investigated forming conditions provide information regarding the influence of the toolpath strategy, vertical step size, and tool-axis orientation on the dimensional accuracy of the formed parts.

From a modeling perspective, the investigated process can be described through a functional relationship between the input parameters and the resulting dimensional deviations. Thus, the bottom level deviation (Δ*h*) and the wall angle deviation (Δ*α*) can be expressed as functions of the main process parameters:

Δ*h* = f(*s*, *tp*, *θ*) (3)

Δ*α* = f(*s*, *tp*, *θ*) (4)

where *s* represents the vertical step size, *tp* the toolpath strategy, and *θ* the tool-axis orientation.

### 3.1. Profile Deviation Along PS1 Section

The first analysis was performed using the PS1 section, which is oriented along the rolling direction of the sheet blank and intersects the axis of the formed part. This section allows the evaluation of the dimensional deviations generated during the incremental forming process.

Figure 7 presents the comparison between the nominal profile of the part and the measured profiles obtained for the investigated forming conditions.

**Figure 8:**
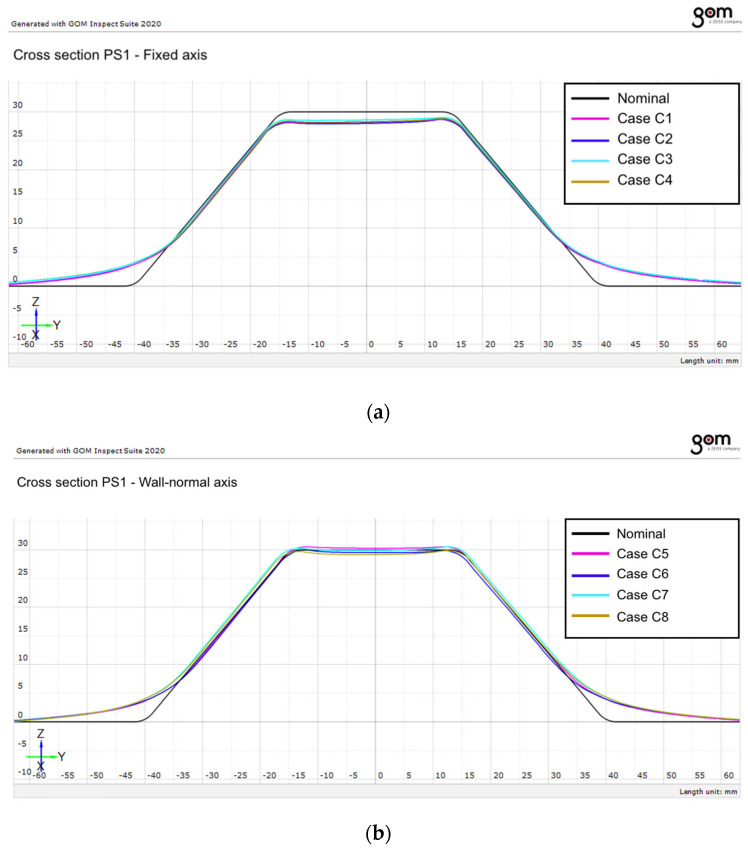
Comparison between the nominal profile and the measured profiles extracted along section PS1 for the investigated forming conditions: (**a**) Fixed axis orientation; (**b**) Wall-angle axis orientation.

As shown in Figure 8, the measured profiles generally follow the shape of the nominal geometry. However, small deviations can be observed along the inclined wall region and near the transition between the wall and the bottom area of the part.

These deviations are typical for the SPIF process and are mainly associated with the elastic springback of the material and with the localized deformation mechanism generated by the incremental forming process.

A comparison between the investigated process parameters indicates that the vertical step size has a significant influence on the actual geometry. Profiles generated using the smaller vertical step size (0.5 mm) tend to follow the nominal geometry more closely compared with those obtained using a step size of 1 mm. This behavior can be explained by the smoother forming process generated by smaller incremental steps.

The influence of the toolpath strategy can also be observed. In general, the spiral toolpath tends to produce profiles that are slightly closer to the nominal geometry compared with the contour toolpath; however, according to the statistical analysis (ANOVA), this difference is not statistically significant under the investigated conditions. This behavior can be attributed to the continuous movement of the punch along the spiral trajectory, which produces a smoother distribution of deformation compared with the discrete contour steps.

In addition to the comparison between the toolpath strategies, the influence of the tool-axis orientation was also evaluated. The results indicate that the use of a wall-normal tool-axis configuration leads to profiles that are closer to the nominal geometry, particularly in the inclined wall region.

### 3.2. Profile Deviation Along PS2 Section

In order to further evaluate the dimensional accuracy of the formed parts, a second cross-sectional analysis was performed using the PS2 plane. This plane is perpendicular to PS1 and is oriented along the transverse direction of the sheet blank.

**Figure 9:**
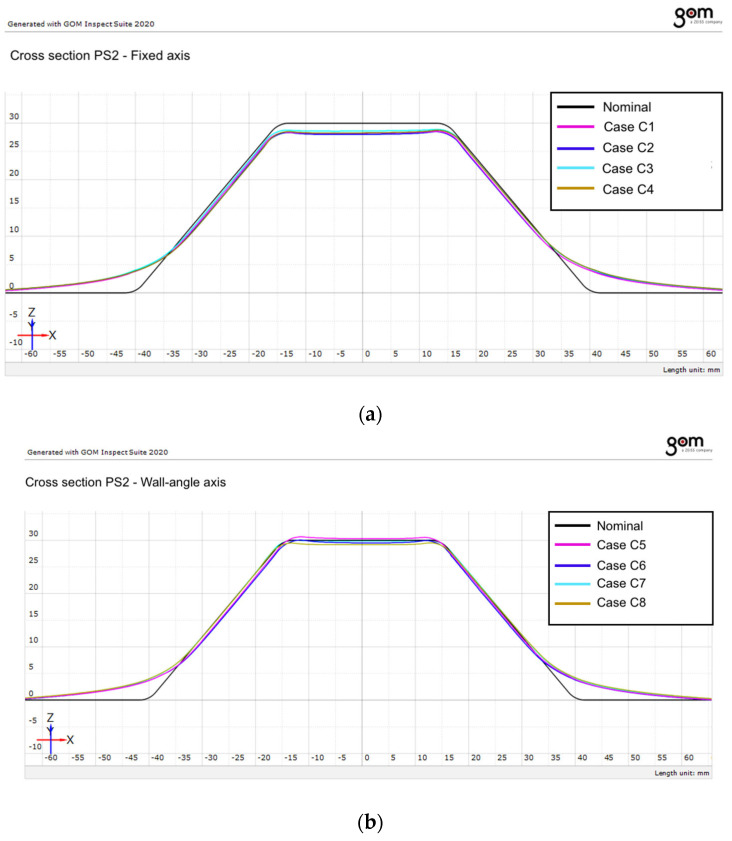
Comparison between the nominal profile and the measured profiles extracted along section PS2 for the investigated forming conditions: (**a**) Fixed axis orientation; (**b**) Wall-angle axis orientation.

The comparison between the nominal geometry and the measured profiles extracted along the PS2 section is presented in Figure 9 for the two investigated tool-axis configurations.

The results obtained for the PS2 section show trends similar to those observed for the PS1 section. The measured profiles generally follow the nominal geometry of the part, although deviations can be observed particularly in the wall region and near the transition between surfaces.

The results indicate that the vertical step size plays an important role in determining the dimensional accuracy of the formed parts. Smaller step sizes lead to profiles that are closer to the nominal geometry, while larger step sizes result in slightly increased deviations.

The comparison between the fixed and wall-normal tool-axis configurations indicates that the wall-normal tool-axis orientation improves the dimensional accuracy of the formed profiles. When the tool axis is oriented perpendicular to the local wall surface, the contact conditions between the tool and the sheet become more favorable, leading to a more uniform deformation of the material.

### 3.3. Bottom Level Deviation (Δh)

In addition to the profile comparison, the deviation of the bottom level of the formed parts was also evaluated. For each investigated forming case, the variation in the bottom height relative to the nominal geometry was determined.

This deviation, denoted as Δ*h*, represents the difference between the measured bottom level of the formed part and the nominal bottom level defined by the CAD model. The value of Δ*h* was measured at the intersection point between the extracted profile and the rotational axis of the conical shape part.

Mathematically, the bottom deviation can be expressed as:

Δ*h* =| *h*_*measured*_ − *h*_*nominal*_| (5)

**Figure 10:**
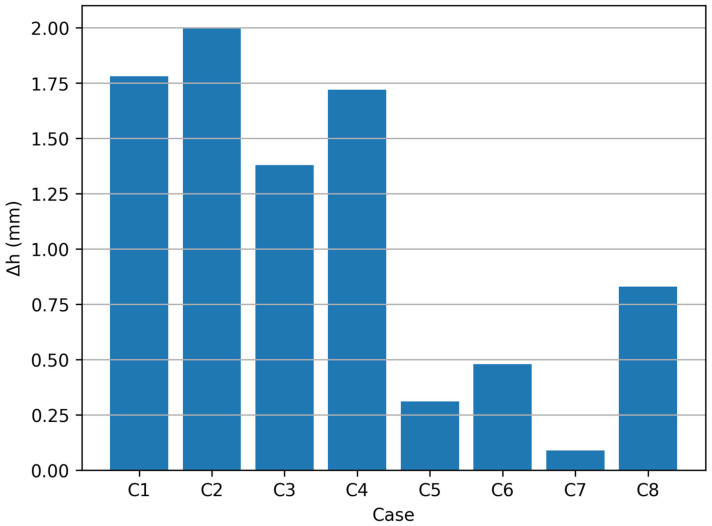
Bottom level deviation (Δ*h*) for the investigated experimental cases.

The obtained values of Δ*h* for all investigated forming cases are summarized in Table 4. Figure 10 shows the bottom level deviation (Δ*h*) for all investigated cases. The lowest values were obtained for the wall-normal tool-axis orientation, especially for case C7, while the largest deviations were observed for the fixed tool-axis configuration.

The results indicate that the bottom deviation is influenced by both the vertical step size and the tool-axis orientation. In general, smaller vertical step sizes lead to reduced deviations of the bottom region of the formed parts. Furthermore, the use of a wall-normal tool-axis configuration contributes to reducing the deviation of the bottom level relative to the nominal geometry.

### 3.4. Wall Angle Deviation

Another important parameter used for evaluating the dimensional accuracy of the formed parts is the wall angle of the part.

**Figure 11:**
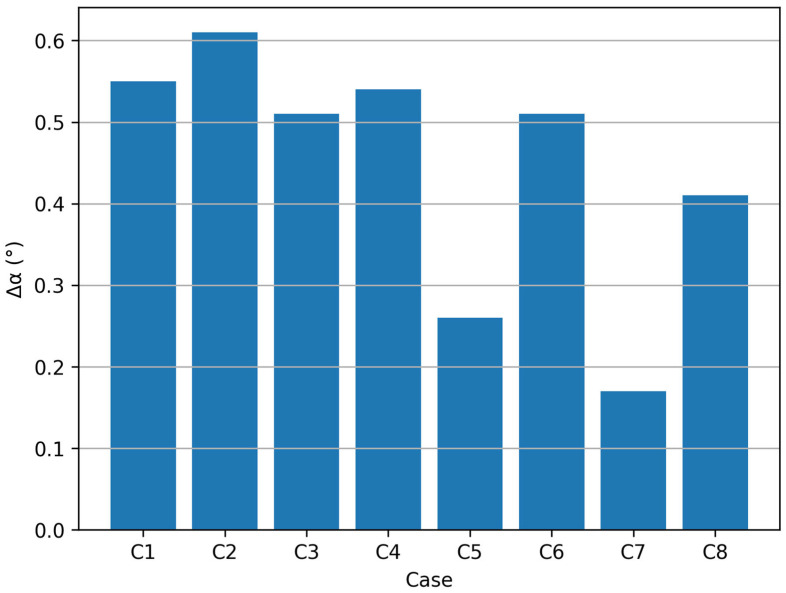
Wall angle deviation for the investigated experimental cases.

For each investigated case, the wall angle was determined from the extracted profiles and compared with the nominal wall angle defined by the CAD model. The obtained results are summarized in Table 5. Figure 11 presents the wall angle deviation for all investigated cases. The smallest wall angle deviation was obtained for case C7, while the largest deviation was observed for case C2.

Mathematically, the wall angle deviation can be expressed as:

Δ*α* = |*α*_*measured*_ − *α*_*nominal*_| (6)

The results show that the actual wall angle is slightly smaller than the nominal value. This behavior is typical for incremental sheet forming processes and is mainly caused by elastic springback and material flow during forming processing.

The comparison between the investigated forming strategies indicates that the wall-normal tool-axis configuration leads to wall angle values that are closer to the nominal geometry.

## 4. Discussion

The experimental results presented in the previous section highlight the influence of several process parameters on the dimensional accuracy of parts produced by RB-SPIF. It should be noted that the compliance of the robotic system may contribute to small deviations in the obtained geometries. However, this effect is systematic and does not alter the comparative trends observed between different process configurations. In particular, the results allow an evaluation of the influence of the toolpath strategy, the vertical step size, and the orientation of the forming tool axis.

The comparison between the measured profiles and the nominal geometry shows that all investigated forming strategies produce geometries that generally follow the target profile. However, noticeable deviations can be observed in specific regions of the part, particularly in the inclined wall region and near the transition between the wall and the bottom area.

These deviations are characteristic of the incremental forming process and are mainly associated with elastic springback, the localized nature of plastic deformation, and the progressive accumulation of positioning errors along the toolpath.

### 4.1. Statistical Analysis Regarding the Effect of Process Parameters on the Dimensional Accuracy of Parts Produced by RB-SPIF

The statistical analysis provides important insights into the influence of the process parameters on the forming accuracy. A multi-factor ANOVA was performed to evaluate the influence of process parameters on the dimensional accuracy of the parts. The statistical analysis was conducted for both the bottom deviation (Δ*h*) and the wall angle accuracy. The *p*-value represents the probability that the observed differences occur by chance. A significance level of 0.05 was adopted, corresponding to a confidence level of 95%. Parameters with *p*-values lower than 0.05 were considered statistically significant. The results indicate that the tool-axis orientation has a statistically significant influence on Δ*h*, while the toolpath strategy does not have a statistically significant effect.

The ANOVA results reveal that the tool-axis orientation is the dominant factor affecting the bottom height deviation (Δ*h*), with a highly significant *p*-value (*p* = 0.001). This behavior can be explained by the change in contact conditions between the tool and the metal blank. When using a wall-normal tool-axis orientation, the contact area becomes more favorable, reducing localized deformation and improving material flow, which leads to lower geometric deviations.

In contrast, the toolpath strategy (circular vs. spiral) does not show a statistically significant influence on Δ*h*. Although spiral trajectories are often associated with smoother deformation due to continuous movement of the tool, in this study their effect is not dominant compared to the tool orientation.

Regarding the wall angle, both the vertical step size and the tool-axis orientation exhibit statistically significant effects. A larger step size increases the incremental deformation per step, which can lead to slight deviations in the final geometry. Meanwhile, the wall-normal tool-axis orientation improves the accuracy by distributing deformation more uniformly.

The lack of statistical significance of the toolpath strategy suggests that, under the tested conditions, geometric accuracy is primarily governed by mechanical interaction (tool orientation and step size) rather than trajectory geometry. This finding is consistent with recent studies emphasizing the importance of tool kinematics and contact mechanics in incremental forming processes [25,26,29]. For example, Bădulescu et al. [25] investigated the influence of innovative and hemispherical tool geometries on SPIF accuracy and surface quality, showing that the geometry of the active contact zone significantly alters frictional behavior and local pressure distribution, which directly affects the fidelity of the formed profile. Similarly, Nagar et al. [26] demonstrated that changes in tool geometry and tool material can improve formed part accuracy even when the global forming strategy remains unchanged, highlighting that local thermo-mechanical interaction plays a more dominant role than trajectory shape alone. A similar conclusion can also be inferred from the work of Ham et al. [29], who showed that the surface topography generated in SPIF is strongly dependent on the local contact mechanics between the tool and the sheet, especially in relation to the sliding behavior of the hemispherical tool tip. These observations support the present finding that, once a stable and continuous trajectory is ensured, dimensional accuracy becomes more sensitive to parameters governing local deformation mechanics than to the selected path type itself.

**Figure 12:**
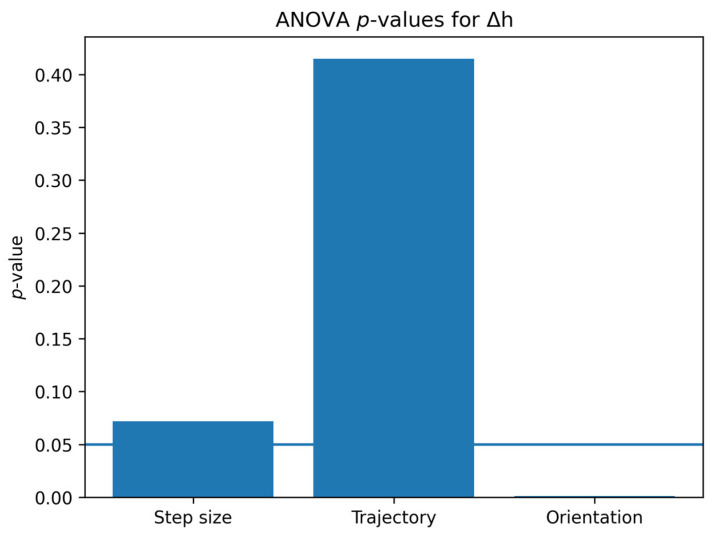
ANOVA *p*-values for Δ*h* showing the influence of process parameters.

**Figure 13:**
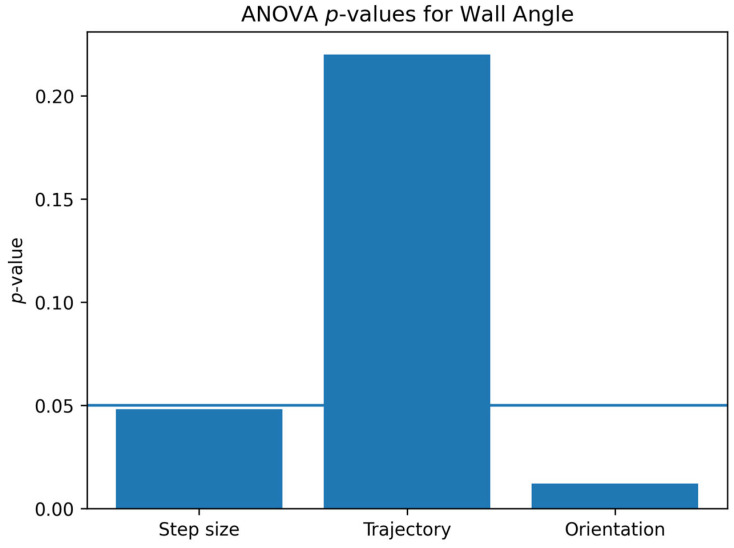
ANOVA *p*-values for wall angle.

The statistical significance was evaluated using a confidence level of 95% (*p* < 0.05). Parameters with *p*-values below this threshold were considered statistically significant. The results are further illustrated in Figure 12 and Figure 13, which present the *p*-values of the analyzed factors.

### 4.2. Influence of the Toolpath Strategy

The experimental results show that the type of toolpath strategy influences the dimensional accuracy of the formed parts. In the investigated cases, two toolpath strategies were analyzed: contour toolpaths and spiral toolpaths.

The spiral toolpath produced slightly better dimensional accuracy compared to the contour strategy under the investigated conditions. A physically grounded explanation for this behavior is related to the continuity of the tool movement. In contour (Z-level) toolpaths, the deformation is applied incrementally at discrete depth levels, leading to local discontinuities in the tool–sheet interaction at each level transition. In contrast, the spiral toolpath represents a continuous variant of the Z-level strategy, providing smoother transitions between successive forming levels. This behavior is also supported by the CAM-based study of Tera et al. [22], where smoother trajectories were associated with improved part accuracy. This continuous deformation reduces local stress concentrations, limits abrupt changes in contact conditions, and contributes to a more uniform material flow.

These observations are consistent with findings reported in the literature, where spiral toolpaths are associated with improved surface finish and smoother deformation evolution due to the exclusion of rapid level transitions. Recent findings reported by Vanhulst et al. [9] and Jung et al. [10], who demonstrated that continuous tool trajectories significantly improve dimensional accuracy by reducing strain localization and eliminating abrupt deformation transitions. In contrast, contour toolpaths introduce discrete forming steps, which generate localized stress concentrations and dynamic effects, leading to increased geometric deviations. This effect is particularly relevant in robotic SPIF systems, where structural compliance amplifies the influence of dynamic loading. As reported by Scholtz et al. [15] and Singh et al. [18], discontinuous tool motion can induce vibrations and path deviations, negatively affecting dimensional accuracy. The continuous nature of spiral toolpaths mitigates these effects, resulting in improved process stability. However, it should be noted that, although the spiral strategy showed slightly improved accuracy, the statistical analysis (ANOVA) indicated that the influence of the toolpath strategy was not significant under the investigated conditions.

For complex geometries, conventional contour and spiral toolpaths may not be sufficient to ensure high dimensional accuracy. In such cases, advanced strategies are required, including feature-based toolpath generation, multi-stage forming approaches, adaptive toolpaths, and workplane rotation methods.

Feature-based and CAM-integrated strategies have been shown to improve toolpath flexibility and geometric control, particularly for parts with varying wall angles [9,10,21]. In addition, Belchior et al. [7] and Behera et al. [8] proposed compensation strategies based on measured or predicted deviations are increasingly used to correct toolpath errors, especially in robotic systems where structural compliance plays a significant role.

Multi-stage forming approaches have also proven effective in improving dimensional accuracy and thickness distribution by reducing strain accumulation and enabling better control of material flow. Vanhulst et al. [9] showed that distributing deformation over multiple forming stages significantly improves thickness homogeneity and reduces localized thinning compared to single-stage strategies. This behavior is attributed to the gradual accumulation of plastic strain, which prevents excessive deformation in critical regions. Similarly, Palwai et al. [17] demonstrated that optimized preform design in multi-stage incremental forming allows a more uniform redistribution of material, leading to improved geometric accuracy and reduced shape deviations. By progressively shaping the geometry, multi-stage strategies minimize abrupt strain gradients and promote stable material flow throughout the forming process. These findings support the interpretation that multi-stage forming not only enhances thickness distribution but also improves dimensional accuracy by stabilizing the deformation process and reducing the risk of localized errors.

Furthermore, recent benchmark studies on complex geometries highlight that geometric accuracy in SPIF is strongly influenced by the interaction between toolpath strategy, process forces, and system compliance [19,20].

These advanced strategies are essential when forming parts with variable wall angles or complex geometrical features, where conventional toolpaths fail to maintain dimensional accuracy. Advanced toolpath strategies are particularly relevant for complex geometries and will be considered in future work.

### 4.3. Influence of the Vertical Step Size

The vertical step size represents a key technological parameter in incremental sheet forming processes. It defines the vertical distance between two consecutive toolpath levels and therefore governs the magnitude of the incremental forming imposed on the sheet during each forming pass. Consequently, the vertical step size significantly influences the deformation mechanism, the strain distribution within the material, and the dimensional accuracy of the formed component.

The results obtained in the present study aligns with recent investigations by Bharti et al. [19] and Lora et al. [30], which confirmed that smaller vertical increments lead to more uniform strain distribution and reduced springback. When a smaller step size is used, the incremental forming process becomes smoother and the resulting surface profile is closer to the nominal geometry. This results in improved geometric accuracy, although at the expense of increased processing time.

Conversely, larger step sizes may increase process productivity but tend to generate larger geometric deviations due to the increased forming applied at each forming increment.

The comparison between the investigated cases indicates that the step size of 0.5 mm generally produces profiles that are closer to the nominal geometry compared with the step size of 1 mm. From the productivity point of view, the use of a smaller vertical step size leads to a higher number of toolpath increments. For the investigated geometry, the 0.5 mm step size requires approximately 60 forming levels, while the 1 mm step size requires about 30 levels. Although the experimental forming time was not recorded directly, this comparison indicates that the smaller step size is associated with a significantly longer processing time. Therefore, a trade-off exists between dimensional accuracy and productivity.

### 4.4. Influence of Tool-Axis Orientation

The orientation of the punch axis represents one of the key aspects investigated in this study. Unlike conventional SPIF processes performed on three-axis CNC machines, robot-based incremental forming systems allow the orientation of the forming tool to be adjusted relative to the geometry of the part [31].

This additional degree of freedom enables the alignment of the tool axis with the local surface normal of the formed geometry. Such an orientation improves the contact conditions between the punch and the sheet surface and may lead to a more favorable distribution of deformation during the forming process. Similar thoughts have been reported by Mohanty et al. [21], who demonstrated that continuous and well-controlled contact between the tool and the sheet significantly improves deformation uniformity and overall process stability in incremental forming. By aligning the tool axis closer to the local surface normal, the contact conditions become more consistent, reducing stress concentration and minimizing deviations from the target geometry.

The experimental results obtained in this study indicate that the wall-normal tool-axis configuration is associated with improved dimensional accuracy compared with the fixed tool-axis configuration. The measured profiles show that the deviations from the nominal geometry are generally smaller when the tool axis is oriented according to the local wall inclination. A plausible explanation is that this configuration improves the local contact conditions between the tool and the sheet blank and promotes a more uniform distribution of forming forces to the material. However, since contact conditions and material flow were not measured directly, this explanation should be regarded as a physically grounded interpretation rather than an experimentally confirmed mechanism.

### 4.5. Identification of the Most Accurate Forming Strategy

A detailed analysis of the experimental results allows the identification of the forming strategy that provides the closest approximation to the nominal geometry.

**Figure 14:**
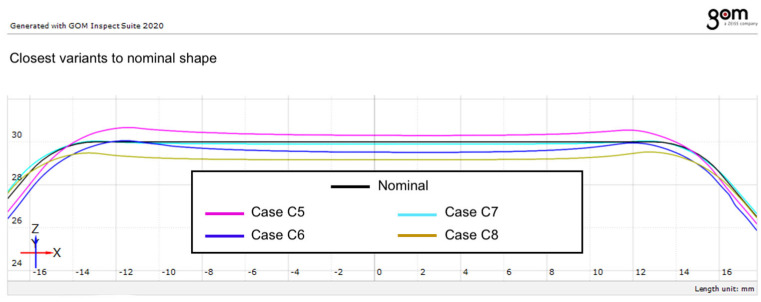
Closest variant of the actual geometry against nominal.

Figure 14 presents a detailed comparison between the nominal profile and the experimentally obtained profiles in the upper region of the formed part. This region is particularly relevant for evaluating the dimensional accuracy because it is susceptible to the so-called pillow effect, a well-known phenomenon in incremental sheet forming. The pillow effect manifests as a slight bulging of the sheet in the central region of the formed part due to the redistribution of stresses and the absence of a supporting die. Consequently, small deviations from the nominal geometry may occur even when the wall profile closely follows the intended shape.

As can be observed in Figure 14, the spiral toolpath combined with a vertical step size of 0.5 mm, experimental case C7, produces the smallest deviation related to nominal geometry. The profile obtained using this strategy follows the nominal contour more closely than the other investigated cases.

This result indicates that the combination of a continuous toolpath strategy and a smaller incremental step contribute to improving the dimensional accuracy of parts produced by robot-based incremental forming.

Furthermore, the results suggest that the use of a wall-normal tool-axis configuration enhances the ability of the process to reproduce the nominal geometry of the part. This observation supports the hypothesis that adaptive tool-axis orientation can represent an effective strategy for improving dimensional accuracy in robot-based incremental sheet forming.

The results obtained in this study show that the dimensional accuracy of robot-based incremental sheet forming can be significantly influenced by the selection of process parameters. In particular, the combination of spiral toolpaths, smaller vertical step sizes, and adaptive tool-axis orientation provides the most favorable conditions for reproducing the nominal geometry of the formed parts.

These findings highlight the potential of robot-based incremental forming systems to implement advanced forming strategies that are difficult to achieve on conventional CNC machines.

## 5. Conclusions

This study investigated the influence of tool-axis orientation on the dimensional accuracy of parts produced by robot-based single point incremental forming. The experimental analysis considered two toolpath strategies (contour and spiral), two vertical step sizes (0.5 mm and 1 mm), and two tool-axis configurations (fixed tool- and wall-normal tool-axis orientation), resulting in eight experimental cases.

Based on the experimental results and the comparative analysis of the measured profiles, the following conclusions can be drawn:

1. The dimensional accuracy of parts produced by RB-SPIF is significantly influenced by the selected process parameters, particularly the tool-axis orientation and, to a minor extent, the vertical step size, as confirmed by the statistical analysis.
2. The vertical step size plays an important role in determining the dimensional accuracy and wall angle. Smaller vertical step sizes (0.5 mm) lead to profiles that follow the nominal geometry more closely, but they also increase the expected processing time because of the larger number of incremental forming levels.
3. Although the toolpath strategy influences the deformation behavior and surface geometry accuracy, the statistical analysis indicates that its effect on dimensional accuracy is not significant under the investigated conditions.
4. The orientation of the forming tool axis has a noticeable influence on the dimensional accuracy of the formed parts. The wall-normal tool-axis configuration improves the contact conditions between the tool and the sheet surface, leading to a more uniform deformation and reduced deviations from the nominal profile. This effect is also confirmed by the statistical analysis.
5. The ANOVA statistical analysis revealed that tool-axis orientation is the dominant statistically significant parameter affecting the bottom height deviation (Δ*h*), while both the vertical step size and tool-axis orientation significantly influence the wall angle. In contrast, the toolpath strategy does not have a statistically significant effect under the investigated conditions.

Overall, the results indicate that adaptive tool-axis orientation represents a promising strategy for improving the dimensional accuracy of parts manufactured by RB-SPIF. The findings of this study contribute to a better understanding of the role of tool-axis orientation in incremental forming processes and highlight the potential of industrial robots for implementing advanced forming strategies.

The conclusions of this study should be interpreted within the limits of the investigated experimental domain. The reported trends are valid for the analyzed part geometry and the specific combinations of process parameters considered in the experimental plan. Further studies are needed to assess whether the same tendencies remain valid for other geometries, materials, thicknesses, and processing conditions.

Future research should focus on the development of adaptive tool-axis orientation strategies in robot-based incremental sheet forming, where the tool orientation is continuously adjusted according to the local geometry of the part. Such approaches could further improve the dimensional accuracy of the formed components and reduce deviations associated with phenomena such as the pillow effect. In addition, integrating numerical simulation with experimental investigations may enable the prediction of geometric deviations and the optimization of process parameters before forming. Further studies should also investigate the influence of tool-axis orientation on forming forces, thickness distribution, and formability limits for different materials and more complex geometries.

## Figures and Tables

**Table 1:** Mechanical properties of DC04 steel.

Material Properties	Value
Thickness	0.67 mm
Young’s modulus	62.589 MPa
Yield strength	161.41 MPa
Ultimate strength	289.98 MPa
Elongation	27.66%
Hardening exponent (*n*)	0.246

**Table 2:** Geometry parameters of the benchmark part used in the experiments.

Parameter	Value
Sheet Blank Material	DC04 Steel
Sheet Blank Thickness	0.67 mm
Sheet Blank Size	250 × 250 mm
Diameter of the large base	80 mm
Total depth	30 mm
Wall angle	50°

**Table 3:** Experimental plan.

Case No.	Wall Angle [°]	Punch Diameter [mm]	Toolpath Strategy	Vertical Step Size [mm]	Tool-Axis Orientation
C1	50	8	Contour	0.5	Fixed
C2			Contour	1	Fixed
C3			Spiral	0.5	Fixed
C4			Spiral	1	Fixed
C5			Contour	0.5	Wall-normal
C6			Contour	1	Wall-normal
C7			Spiral	0.5	Wall-normal
C8			Spiral	1	Wall-normal

**Table 4:** Bottom level deviation (Δ*h*) relative to the nominal geometry.

Case No.	Wall Angle [°]	Toolpath Strategy	Vertical Step Size [mm]	Tool-Axis Orientation	Bottom Level Deviation (Δ*h*) [mm]
C1	50	Contour	0.5	Fixed	1.78
C2		Contour	1	Fixed	2
C3		Spiral	0.5	Fixed	1.38
C4		Spiral	1	Fixed	1.72
C5		Contour	0.5	Wall-normal	0.31
C6		Contour	1	Wall-normal	0.48
C7		Spiral	0.5	Wall-normal	0.09
C8		Spiral	1	Wall-normal	0.83

**Table 5:** Wall angle deviation.

Case No.	Wall Angle [°]	Toolpath Strategy	Vertical Step Size [mm]	Tool-Axis Orientation	Measured Wall Angle [°]	Deviation [°]
C1	50	Contour	0.5	Fixed	49.45	0.55
C2		Contour	1	Fixed	49.39	0.61
C3		Spiral	0.5	Fixed	49.49	0.51
C4		Spiral	1	Fixed	49.46	0.54
C5		Contour	0.5	Wall-normal	49.74	0.26
C6		Contour	1	Wall-normal	49.49	0.51
C7		Spiral	0.5	Wall-normal	49.83	0.17
C8		Spiral	1	Wall-normal	49.59	0.41

## Data Availability

The original contributions presented in the study are included in the article, further inquiries can be directed to the corresponding author.
